# Nurses’ job crafting, work engagement, and well-being: a path analysis

**DOI:** 10.1186/s12912-023-01573-6

**Published:** 2023-10-30

**Authors:** Sujeong Han

**Affiliations:** College of Nursing, Konyang University, 158 Gwanjeodong-ro, Seo-gu, Daejeon, 35365 Republic of Korea

**Keywords:** Nurses, Job crafting, Well-being, Relational crafting, Task crafting, Work engagement

## Abstract

**Background:**

Nurses’ well-being is a topic of interest at both individual and organizational levels. Studies that explore the relationship between nurses’ job crafting, work engagement, and well-being are scarce. Therefore, the purpose of this study was to confirm the effect of job crafting and work engagement on nurses’ well-being.

**Methods:**

This cross-sectional survey study involved 207 nurses within a week in July 2022 across two centers, among whom the response rate was 99%. Three following instruments were used in the survey: the 14-item well-being scale, the 12-item job crafting scale, and the 9-item Utrecht Work Engagement Scores (UWES). Path analysis was performed and goodness of fit was evaluated.

**Results:**

Job crafting and work engagement were strongly correlated with well-being, and nurses’ well-being was affected by job crafting and work engagement. Path model fit indices were adequate. The mediating effect of work engagement on the relationship between job crafting and well-being revealed that task job crafting influenced psychological well-being through work engagement (Effect: 0.15, 95% confidence interval [CI]: 0.08–0.22, p = 0.001). Furthermore, relational job crafting influenced social well-being through work engagement (Effect: 0.22, 95% CI: 0.11–0.38, p = 0.001).

**Conclusion:**

The study’s findings can help strategize human resource management programs to enhance relational job crafting to improve nurses’ social well-being and enhance task job crafting to improve their psychological well-being. Furthermore, through job crafting, improved human resource policies can enhance work engagement and improve nurses’ well-being.

## Background

The well-being of our nurses is among the most important considerations in providing excellent patient care. To achieve the best possible outcomes for patients and their families, nurses need to work at the highest level of well-being. Well-being is an ambiguous concept with diverse definitions by researchers. The concepts used interchangeably with well-being include happiness, quality of life, satisfaction, and enjoyment [1]. Yet, the establishment of a specific definition is challenging owing to the diverse perspectives and societal values concerning well-being. Nevertheless, three principal theories and conceptual definitions exist, including emotional well-being, a state of life satisfaction and positive emotions; psychological well-being, a self-assessment of the ability to navigate through social interactions, make autonomous decisions, and maintain a sense of control over external circumstances, and social well-being, a self-evaluation of the quality of life [2]. Keyes [3] attempted to integrate these three concepts into a single mental health concept and presented the conditions for a fulfilled life, focusing on emotional, social, and psychological well-being.

Lee [4] and San [5] et al. revealed that nurses’ well-being is related to personal factors, job characteristics, and the work environment. San [5] categorized the factors affecting nurses’ well-being into personal and work and concluded that personal characteristics are antecedents of nurses’ well-being. Furthermore, when nurses are well, the outcome variables include improved work performance, fulfillment, health, and reduced burnout, thereby improving productivity and patient outcomes. In other words, their well-being relates to job satisfaction as well as their feelings of overcoming and enduring personal conflict. As work-related variables influence their well-being, considering these factors from their job’s perspective is necessary to assess their well-being.

From a job perspective, individual and organizational well-being increases with the meeting of the employees’ basic needs for autonomy, belongingness, and passion [6]. Furthermore, nursing outcomes such as quality of care and nursing performance are closely related to well-being [7]. The well-being of employees is enhanced as their basic needs are met through job crafting, which is the aligning of work ownership to their values or pleasures to perform better, experience excitement, and derive purpose from work [6, 8]. Previous studies have revealed the positive effect of job crafting on nurses’ well-being [9, 10]. Employees with high job crafting levels experience positive emotions, such as well-being, enjoyment, and enthusiasm, along with improved psychological and physical health [11], leading to complete well-being [12]. Therefore, it was hypothesized that job crafting for nurses would have a positive effect on their well-being (Hypothesis 1, H1).

Job crafting includes changing what you do as part of your job, one’s approach to work, along with interaction with others. Furthermore, job crafting takes the form of increasing one’s challenges on the job, resources, and ability to reduce the demands of the job that interfere with one’s own [13]. When nurses decide to balance their job resources and demands, they become more productive, the motivation of which increases their job engagement [14]. Accumulating evidence suggests that, among nurses, high levels of job crafting are associated with high levels of work engagement [13–15]. Therefore, it was hypothesized that nurses’ job crafting would positively affect work engagement (H2).

Work engagement, being active and vigorous, reflects the qualities of vital workability and professional identity, which involves dedication (loving work and a sense of honor), vigor (high vitality and resilience at work), and absorption (dedication to work and reluctancy to put work aside) [16]. Further, Ding et al. have revealed that work engagement is crucial to achieving nurses’ subjective well-being [16]. Another previous study revealed work engagement’s significant direct effect on mental well-being [17, 18]; all these studies led us to predict that work engagement would positively affect nurses’ well-being (H3).

**Fig. 1:**
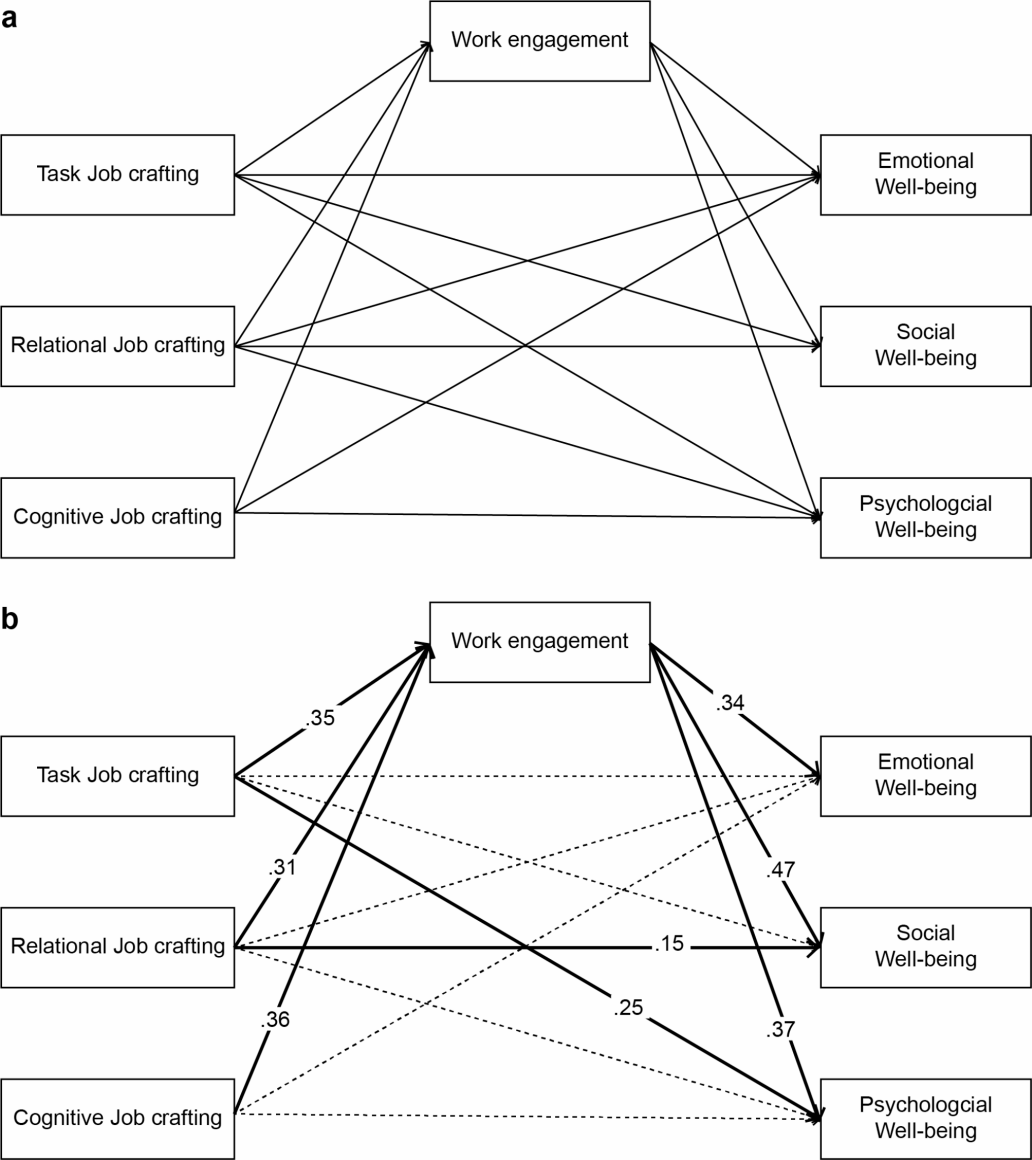
Theoretical framework and final path model (**a**) Hypothesized model of the study; (**b**) Path coefficients of variables in the path model

Taken together, nurses’ levels of job crafting can help predict the components that influence work engagement, ultimately affecting their well-being. The three sub-domains of job crafting include task, relational, and cognitive. To date, no study has examined their effects, nor the effects of work engagement on nurses’ well-being. Therefore, this study investigated the effects of task, relational, and cognitive job crafting on well-being and work engagement, which respectively are sub-domains of emotional, social, and psychological well-being. the mediating effect of work engagement was identified through a path analysis (Fig. 1a).

## Methods

### Hypotheses

The following hypotheses were tested:

H1. Job crafting factors (task, relational, and cognitive) are positively related to well-being factors (emotional, social, and psychological, respectively).

H2. Job crafting factors (task, relational, and cognitive) are positively related to work engagement.

H3. Work engagement is positively related to well-being factors (emotional, social, and psychological).

### Study design

The study adopted a cross-sectional design. For this path analysis, a hypothetical path model was constructed and tested to examine the relationships among the nurses’ job crafting, work engagement, and well-being.

### Participants & data collection

This study included nurses working at two general hospitals in cities D and G. There are approximately 1,200 nurses in these two general hospitals. The study’s participant selection criteria were based on convenience sampling among nurses who had worked for at least six months in their current department (considering the time required to familiarize themselves with the work in the wards); nurses with no work experience or those who worked for less than 6 months were excluded. The survey received approval from the hospital’s director of nursing. The data were collected between July 11 and July 17, 2022. The notice on recruitment of research subjects for the survey was displayed on the bulletin board of the nursing department by the nurse in charge of each hospital. The pen and pencil questionnaires were distributed to nurses. After the completion of the survey period, some compensation was provided to the participants who submitted their consent to the survey and answered all questions in the survey. The number of participants required for this study was based on the perspective [19] that path analyses should have at least ten participants per predictor variable, ideally with 200 individuals. Thus, data from 211 participants were collected. The questionnaire was completed in approximately 10 min. Of the 211 distributed questionnaires, 209 were returned, among which two were incomplete and 207 proceeded for analysis.

### Measures

#### Well-being

Keyes [3], who integrated the concepts of emotional, social, and psychological well-being into one comprehensive concept, developed the “well-being” measurement tool. Lim et al. [2] selected a tool that has been validated for the general Korean public. The tool consists of 14 questions measuring social (5 questions), psychological (6), and emotional (3) well-being. It uses a 6-point Likert scale of 0 (nothing) to 5 (every day) for each question. In a study by Lim et al. [2], the overall reliability was 0.93, emotional well-being was 0.88, social well-being was 0.81, and psychological well-being was 0.90. This study’s overall reliability was 0.94, and the internal reliability of the emotional, social, and psychological well-being subscales were 0.93, 0.85, and 0.91, respectively.

#### Job crafting

Kim and Shim [20] modified Ghitulescu’s [21] job crafting measurement tool into 12 questions to suit the Korean context. This tool consists of three subdomains: task job crafting, which involves the members of the organization making physical changes to their work; relational job crafting, to alter the social interactions with their colleagues; and cognitive job crafting, which involves reconstructing their work’s meaning and identity. This tool uses the 5-point Likert scale of 1 (not true) to 5 (very true), with higher scores indicating a higher degree of job crafting. This tool was reported to have concurrent, convergent, and construct validity [20]. The overall reliability was 0.93, and the subscales were: task job crafting, 0.90; relational job crafting, 0.93; cognitive job crafting, 0.94.

#### Work engagement

The Utrecht Work Engagement Scale by Schaufeli et al. [22] was translated into Korean by Chang [23]. The reliability and convergent validity of the study were demonstrated in a previous study [15]. This tool has nine questions in three sub-areas, with three questions each on vitality, commitment, and immersion. This tool uses a 5-point Likert scale, with higher scores indicating higher work engagement. In Chang’s study [23], the reliability was 0.92, while in this study, it was 0.95.

### Validity and reliability

The validity and reliability of the scales adopted in the study were tested according to the method described by Sartori and Pasini [24]. The well-being score, Cronbach’s alpha, in this study was 0.94. Deleting items one by one decreases the total reliability of the scale; hence, the reliability of the items was verified. Accordingly, the items’ total correlation ranged from 0.31 to 0.82. Moreover, the job crafting scale score, Cronbach’s alpha, in this study was 0.93. Similar to the well-being scale, deleting items one by one decreases the total reliability of the scale; hence, the reliability of the items was verified. The items’ total correlations ranged from 0.35 to 0.81. Lastly, the UWES Cronbach’s alpha was 0.95, and after verifying the reliability of the items, the correlation ranged from 0.48 to 0.84. Thus, the reliability of the scales used was established. Furthermore, confirmatory factor analysis was performed, and the fit indexes were root mean square error of approximation (RMSEA) = 0.065 (95% CI: 0.049–0.081), comparative fit index (CFI) = 0.971, and goodness of fit index (GFI) = 0.909. Thus, construct validity was confirmed. No items were determined to be dropped or excluded from the measure.

### Ethical consideration

The study was approved by the researcher’s institutional review board (KYU-2022-05-015-001). The information sheet provided to potential participants explained the purpose of the study, the questionnaire process, the anonymous processing of written consent forms, and the freedom to withdraw at any time without any disadvantages. Participants were informed of data confidentiality and that the data would be managed according to the personal information protection principles and destroyed at the end of the study. All study participants who provided consent before completing the questionnaire were included, and those who did not were excluded.

### Statistical analysis

SPSS/WIN 22.0 and AMOS 18.0 (IBM Corp., Armonk, NY, USA) were used for the data analysis. The participants’ general characteristics were subjected to technical statistical analysis. The correlation between the research variables was confirmed using Pearson’s correlation coefficient. Univariate normality was confirmed via skewness and kurtosis. The values of skewness ranged from − 0.95 to 0.00, and those of kurtosis from − 0.36 to 2.52; both satisfied the conditions for normality. Multivariate normality was confirmed using multivariate kurtosis = 9.87 and critical ratio = 8.49, which did not meet Mardia’s criteria [25]. However, this study’s data were subjected to path analysis using the maximum likelihood estimation method, in accordance with a report [26] in which univariate normality was met (Table 1). The estimated parameter was reliable using the maximum likelihood method, even if the multivariate normality assumption was not met. The goodness of fit of the model was assessed using the chi-square (x^2^) test, RMSEA, GFI, and CFI, which are absolute goodness of fit indices. Generally, if the probability of significance of the chi-squared value was above the significance level (0.05), the model was adequate. The model was considered good if the RMSEA was below 0.08 (with significance assessed by a two-tailed p-value). Additionally, if the model fit index GFI was 0.90 and CFI was ≥ 0.95, it was a good model [25]. The research hypotheses were tested by performing a path analysis model with a maximum likelihood estimator. Bootstrapping was conducted using 5000 bootstrap samples, along with 95% bias-cleaned confidence intervals (CI), to assess the significance of indirect effects.

**Table 1:** Correlation among variables (*N* = 207)

Variables	Min.	Max.	Skewness	Kurtosis	M ± SD	1	2	3	4		5	6	7
						R (*p-*value)							
1. Task JC	4.00	20.00	-0.57	0.74	13.69 ± 3.14	1							
2. Relational JC	4.00	16.00	-0.39	1.11	11.92 ± 2.27	0.57 (< 0.001)	1						
3. Cognitive JC	4.00	20.00	-0.95	2.52	15.54 ± 2.93	0.49 (< 0.001)	0.51 (< 0.001)	1					
4. WE	0.00	54.00	0.00	0.03	26.60 ± 10.12	0.61 (< 0.001)	0.59 (< 0.001)	0.49 (< 0.001)		1			
5. Emotional W.	0.00	13.00	-0.40	-0.36	7.40 ± 3.03	0.35 (*p* < 0.001)	0.33 (*p* < 0.001)	0.33 (*p* < 0.001)		0.46 (*p* < 0.001)	1		
6. Social W.	0.00	24.00	-0.15	-0.08	11.53 ± 4.41	0.40 (*p* < 0.001)	0.44 (*p* < 0.001)	0.31 (*p* < 0.001)		0.57 (*p* < 0.001)	0.64 (*p* < 0.001)	1	
7. Psychological W	0.00	30.00	-0.44	0.47	16.46 ± 5.35	0.56 (*p* < 0.001)	0.49 (*p* < 0.001)	0.40 (*p* < 0.001)		0.61 (*p* < 0.001)	0.70 (*p* < 0.001)	0.78 (*p* < 0.001)	1

Abbreviations: M, mean; Max, Maximum; Min, Minimum; JC, Job crafting; SD, standard deviation; WE, Work engagement; W, Well-being

## Results

### Characteristics of respondents

The average age of the respondents was 33.5 years, and most (93.2%) were females. Of these, 62.8% were unmarried, 69.1% were university graduates, and 80.2% were general nurses. Of the respondents, 31.4% and 36.7% worked in general and special wards, respectively. In total, 37.7% had over ten years of hospital experience, followed by 27.1% with 5–10 years. Their average work experience was 8.9 years (± 6.88) (Table 2).

**Table 2:** Participants’ general characteristics (*N* = 207)

Characteristics	Categories	n (%) or M ± SD	
Age (year)		33.5 ± 8.67	
Sex	F	193	(93.2)
	M	14	(6.8)
Marital status	Single	130	(62.8)
	Married	77	(37.2)
Educational degree	Associate degree	44	(21.3)
	Bachelor’s degree	143	(69.1)
	≧Master degree	20	(9.7)
Current work unit	General unit	65	(31.4)
	Special unit*	76	(36.7)
	Integrated nursing care service	31	(15.0)
	Others	35	(16.9)
Position	Staff nurse	166	(80.2)
	≥Charge nurse	41	(19.8)
Clinical career at the current hospital (year)	< 2	28	(13.5)
	2-<5	45	(21.7)
	5-<10	56	(27.1)
	≥ 10	78	(37.7)
		8.97 ± 6.88	

* Special unit, operating room; intensive care unit; artificial kidney room

Abbreviations: M, mean; SD, standard deviation

### Descriptive statistics and correlations of the variables

The participants’ scores for each sub-domain job crafting were 13.69 ± 3.14, 11.92 ± 2.27, and 15.54 ± 2.93 points for task, relational, and cognitive job crafting, respectively, out of a total score of 20 points. Work engagement averaged 26.60 ± 10.12 points out of 54, well-being was 7.40 ± 3.03 out of 15, social well-being was 11.53 ± 4.41 out of 25, and psychological well-being was 16.64 ± 5.35 out of 30 points. In bivariate correlation analysis, all three sub-domains of job crafting correlated positively with work engagement (r = 0.49–0.61, *p* < 0.001 for all), and the three sub-domains of well-being (r = 0.31–0.56, *p* < 0.001 for all). Work engagement correlated positively with the three sub-domains of well-being (r = 0.46–0.61, *p* < 0.001 for all) (Table 1).

### Goodness of fit of the model and path analysis

In the suitability test of the research model on the relationship between job crafting, work engagement, and nurses’ well-being, the theoretical model appeared saturated (x^2^ = 0.00; df = 0; x^2^/df = none; RMSEA, none; CFI, 1.00; GFI, 1.00). Therefore, we considered modifying the model [27]. First, the model’s fit index was confirmed by sequentially setting the covariance of the high index on the well-being subscale as the corrected index value. When the model’s fit was assessed by removing insignificant paths, it was best to remove the path from cognitive crafting to social well-being. The model’s goodness of fit was x^2^ = 0.01 (*p* = 0.908); x^2^/df, 0.01; RMSEA, 0.00; CFI, 1.00, and GFI, 1.00. The direct, indirect, and overall effects of the main variables related to the nurses’ well-being are presented in Table 3.

**Table 3:** Standardized direct, indirect, and total effects of variables in the modified model

Endogenous variables	Exogenous variables	Direct effects(*p*-value)		Indirect effects(*p-*value)		Total effects(*p*-value)		R^2^ (SMC)
Work engagement	Task JC	0.35 (0.001)				0.35 (0.001)		0.473
	Relational JC	0.31 (0.001)				0.31 (0.001)		
	Cognitive JC	0.16 (0.045)				0.16 (0.045)		
Emotional well-being	WE	0.34 (0.001)				0.34 (0.001)		0.406
	Task JC	0.06 (0.441)		0.12 (0.001)		0.18 (0.005)		
	Relational JC	0.04 (0.788)		0.10 (0.001)		0.14 (0.115)		
	Cognitive JC	0.13 (0.112)		0.05 (0.045)		0.18 (0.018)		
Social well-being	WE	0.47 (0.001)				0.47 (0.001)		0.406
	Task JC	0.02 (0.715)		0.17 (0.001)		0.19 (0.013)		
	Relational JC	0.15 (0.068)		0.14 (0.001)		0.29 (0.001)		
	Cognitive JC			0.08 (0.045)		0.08 (0.045)		
Psychological well-being	WE	0.37 (0.001)				0.37 (0.001)		0.406
	Task JC	0.25 (0.001)		0.13 (0.001)		0.38 (0.001)		
	Relational JC	0.11 (0.168)		0.11 (0.001)		0.12 (0.002)		
	Cognitive JC	0.05 (0.357)		0.06 (0.045)		0.11 (0.060)		
			Estimate		[95% CI]		*p-value*	
Effect 1: Task JC → Work engagement → Psychological well-being			0.147		[0.08, 0.22]		0.001	
Effect 2: Relational JC → Work engagement → Social well-being			0.224		[0.11, 0.38]		0.001	

Abbreviations: CI, Confidence interval; JC, Job crafting; SMC, Squared multiple correlations; WE, Work engagement

In the direct effect path, among the effects of job crafting on well-being, only the effect of task crafting on psychological well-being (β = 0.25, *p* = 0.001) was statistically significant, while the effects of relational crafting and cognitive crafting on well-being (emotional, social, and psychological) were not statistically significant, partially confirming H1. The significant effects of task crafting (β = 0.35, *p* = 0.001), relationship crafting (β = 0.31, *p* = 0.001), and cognitive crafting (β = 0.16, *p* = 0.045) on work engagement, confirmed H2. Finally, there were significant effects of work engagement on emotional well-being (β = 0.34, *p* = 0.001), social well-being (β = 0.47, *p* = 0.001), and psychological well-being (β = 0.37, *p* = 0.001), thus confirming H3 (Fig. 1-B).

Considering the indirect effects of significant variables, task crafting indirectly affected emotional well-being through work engagement (β = 0.12, *p* = 0.001), social well-being (β = 0.17, *p* = 0.001), and psychological well-being (β = 0.13, *p* = 0.001). Relational crafting indirectly affected emotional well-being (β = 0.10, *p* = 0.001), social well-being (β = 0.14, *p* = 0.001), and psychological well-being (β = 0.11, *p* = 0.001) through work engagement. Cognitive crafting indirectly influenced emotional well-being (β = 0.05, *p* = 0.045), social well-being (β = 0.08, *p* = 0.045), and psychological well-being (β = 0.06, *p* = 0.045) through work engagement.

Examining the mediating effect of work engagement on the relationship between job crafting and well-being revealed that the former affected psychological well-being (effect = 0.15, 95% confidence interval [CI] = 0.08, 0.22, *p* = 0.001), and relational crafting affected social well-being (effect = 0.22, 95% CI = 0.11, 0.38, *p* = 0.001) through work engagement.

The explanatory power of the significant impact of task, relational, and cognitive job crafting on work engagement was 47.3%, and those of the sub-domains of job crafting and work engagement on emotional, social, and psychological well-being were 40.6%, 40.6%, and 40.61%, respectively (Table 3).

## Discussion

This study found that work engagement and job crafting influenced nurses’ well-being, which is consistent with previous studies. Positive job crafting increases work engagement, which leads to a higher level of well-being [14, 28–31]. Specifically, task crafting directly influenced psychological well-being. Psychological well-being is based on the evaluation of self-acceptance, autonomy, environmental control, and personal growth [3]. These findings can be interpreted as the nurses’ efforts to change the boundaries of work, such as the hours, content, and order of work, providing them with a sense of psychological well-being. In other words, the high level of work crafting could have physically adapted to their work, growth, autonomy, and environmental control, resulting in psychological well-being. Furthermore, in previous studies [9], job crafting was found to be a predictor of psychological well-being, especially when nurses engaged in job crafting to improve their job resources and demands [10]. Therefore, nurse managers should take appropriate measures to support nurses’ job resources, both structurally and socially, and challenge the job demands, while learning about new technologies and approaches to nursing.

Another notable finding of this study is that when nurses engage in relational crafting behaviors that change their social interactions at work, their levels of social well-being increase. When individuals have a sense of belonging to the community, their contribution to and acceptance and understanding of society, facilitate social well-being [3]. Therefore, the improved work-related social interaction of the nurses correlates with a higher level of social well-being, as they have a sense of belonging to the community and recognition. A previous study defined emotional well-being as having both positive and negative emotions and examined its relationship with job crafting. The authors confirmed that job crafting led to emotional well-being [28]. This is consistent with the findings of the current study. Job crafting also affected positive emotions when the nurse pursued job resources and reducing job demands for better job crafting resulted in more positive emotions [29]. Another study has also examined the effect of job crafting on the well-being of doctors and nurses, and the well-being and performance of both professionals increased with job crafting. In particular, for nurses, pursuing challenges or job crafting with reduced demands did not affect well-being, whereas pursuing job resources positively affected well-being, similar to the results of this study [30]. In summary, nurses who initiate job crafting have more positive job attitudes, which is a more positive self-evaluation that increases social and psychological well-being. Therefore, nurse managers should recognize and apply job crafting for the well-being of their members.

In this study, all sub-domains of job crafting positively affected work engagement. These findings have also been confirmed in previous studies of nurses, as they seek more job resources, and their job attitudes become more positive and fulfilling (work engagement) [14]. Once members maintain an optimal balance between job resources and demand through job crafting, continuous work engagement can be created by altering the tasks, meaning, and identity [31, 32]. Previous studies have shown that empowering leadership positively affects job crafting, which increases the nurses’ structural and social resources and increases challenging demands. Empowering leadership particularly affects work engagement by increasing the nurses’ cognitive crafting [10]. Therefore, as a strategy to increase nurses’ work engagement, it is necessary to develop leadership, so that they coach their colleagues and activate nurses’ job crafting.

The results also showed that work engagement positively affected all three sub-domains of well-being, that is, emotional, social, and psychological well-being increased when nurses were engaged in their work. Studies with similar findings [16, 17, 28, 29] observed that increased work engagement led to well-being. Tomieeto et al. [33] found that the impact of sub-domains of work engagement on nurses’ well-being differed according to the age of the nurse. They also reported differences in the impact of work engagement on well-being due to cultural differences between countries. Therefore, there is a need to further explore the relationship between work engagement and well-being by considering nurses’ characteristics, such as age and cultural background.

In particular, the current study confirmed the mediating effect of work engagement, especially between task and relational crafting, and psychological and social well-being. In a previous study [28], employees’ job crafting helped improve their work engagement and their high work engagement contributed to health improvements. This was also consistent with the findings for nurses [18]. In terms of implications for nursing management, the results of the current study suggest that helping activate nurses’ work and relationship crafting can increase the nurses’ vitality, commitment, and absorption in their work, to eventually improve their psychological and social well-being. Through the association found in this study, it can be inferred that job crafting and job enthusiasm are closely related to the nurses’ work environment.

### Limitation

This study is significant because it reveals the association between work engagement and well-being in each job crafting sub-domain, categorizing well-being as per the emotional, social, and psychological factors, and presents theoretical and practical results. However, this study’s results should be interpreted with consideration of its limitations. First, this was a cross-sectional study; therefore, causal relationships between the variables could not be established. Secondly, self-reported data may be susceptible to denial and social desirability biases. Finally, the nurses in this study were recruited from two hospitals using convenience sampling, which raises generalization concerns. Thus, further research should include a larger sample size and recruit participants from different hospitals to ensure representative results. Further research should identify different variables affecting nurses’ well-being and provide evidence-based resources to improve it. Job crafting and work engagement intervention programs need to be developed. Further research is needed to confirm the impact of the program on the nurses’ well-being.

## Conclusion

Job crafters are known to have high levels of well-being, provide better care, and help hospitals achieve their organizational goals. This study examined the effects of job crafting and work engagement on nurses’ well-being. Job crafting affects the workers’ engagement, which in turn affects their well-being. Task job crafting influenced psychological well-being through work engagement, and relationship job crafting influenced social well-being through work engagement, both confirming the mediating effect of work engagement in the relationship between job crafting and well-being. When applying the results of this study to clinical practice, nurse managers should be aware that changes in work boundaries affect the psychological well-being of staff nurses and changes in social interactions at work affect social well-being. Therefore, they should adopt strategies such as identifying the facilitators and barriers to job crafting for staff nurses, ensuring education and training opportunities to promote job crafting, and providing intervention programs for hospital nurses.

## Data Availability

Data cannot be shared publicly due to restrictions imposed by the Konyang University Institutional Review Board. Data are available from the Konyang University Institutional Data Access/Ethics Committee for researchers who meet the criteria for access to confidential data. Data requests can be addressed to the Konyang University Institutional Review Board (82-42-600-8466, kirb@konyang.ac.kr).

## Abbreviations

CFI: Comparative fit index
GFI: Goodness of fit index
RMSEA: Root mean square error of approximation
